# *Bacillus subtilis* RBT-7/32 and *Bacillus licheniformis* RBT-11/17 as New Promising Strains for Use in Probiotic Feed Additives

**DOI:** 10.3390/microorganisms11112729

**Published:** 2023-11-08

**Authors:** Vera Yaderets, Nataliya Karpova, Elena Glagoleva, Alexandra Shibaeva, Vakhtang Dzhavakhiya

**Affiliations:** Laboratory of Biotechnology of Industrial Microorganisms, Department of Biotechnology and Technology of Bioorganic Synthesis Products, Russian Biotechnological University (ROSBIOTECH), Moscow 125080, Russia; ashatanr@mail.ru (N.K.); glagolevaev@mail.ru (E.G.); aleksandrashibaeva@mail.ru (A.S.)

**Keywords:** *Bacillus subtilis*, *Bacillus licheniformis*, probiotic strains, feed additive, antagonistic activity

## Abstract

The normal functioning of a gastrointestinal microflora in poultry and livestock is of significant importance, since its imbalance negatively influences an organism’s functions. In this study, the UV mutagenesis and selection were used to obtain two *Bacillus* strains possessing antagonistic activity towards *Escherichia coli* and *Staphylococcus aureus*, and their potential as a probiotic feed additive was evaluated. Compared to the parental strains, the ability of *B. subtilis* RBT-7/32 and *B. licheniformis* RBT-11/17 strains to suppress *E. coli* increased by 77 and 63%, respectively; the corresponding ability of these strains to suppress *S. aureus* increased by 80 and 79%, respectively. RBT-11/17 could not utilize microcrystalline cellulose and carboxymethyl cellulose, whereas cellulolytic activity of RBT-7/32 was doubled compared to the initial strain. The amylolytic activity of new strains was increased by 40%. Cultivation of strains on media containing soybean, pea, and corn meal did not provide any difference in the biomass production compared to the control. The heating of a water suspension of a dried biomass of the strains for 10–20 min at 80 and 100 °C or incubation in water solutions of citric, ascorbic, acetic, and formic acids (pH 3.0) for 3 and 24 h at 40 °C did not provide any negative influence on the spore survivability. Both strains were evaluated for their resistance to a number of veterinary antibiotics. Thus, RBT-7/32 and RBT-11/17 strains have good prospects for use in feed additives.

## 1. Introduction

Taking into account the current pace of development of agriculture in Russia, the provision of agricultural industry with high-quality and balanced forage and feed additives is of great importance, because the quality of feeding of livestock and poultry is one of the main factors directly influencing on their growth and development and ensuring the optimal use of a genetic potential of their productivity [1,2,3]. Along with the physiological importance of a balanced nutrition, the normal functioning of gastrointestinal microflora is of great significance, since its imbalance negatively influences on various functions of the organism [1,4,5,6].

Gastrointestinal (GI) diseases represent one of the main causes of death of young animals taking the second place after viral diseases [7]. GI diseases are primarily associated with disorders of the intestinal biocenosis, and a resistance decrease caused by a weakening of the immune system under conditions of overcrowding of the poultry and livestock within limited areas, technological and feed stresses, and chemical stresses caused by the use of antibacterial drugs and antibiotics [8]. In the most cases, animal and poultry farms use significant amounts of antibiotic drugs for therapeutic and prophylactic purposes. However, a long-term use of antibiotics (especially broad-spectrum ones) results in the development of drug resistance in pathogenic and opportunistic microflora [9,10,11].

In recent years, some new approaches for the treatment of GI diseases have been developed. These approaches are based on the recovery of the natural microflora using so-called probiotics, biologically active products, in which effectiveness is not inferior to some antibiotics and chemotherapeutic agents [12,13]. When selecting potential probiotic microorganisms, one should take into account a number of their functional, safety, and technological characteristics. Probiotic bacteria should satisfy the following main requirements: they should be non-pathogenic and non-toxic, possess antagonistic activity against pathogenic and opportunistic microflora, and remain viable both in the gastrointestinal tract (GIT) and during storage in a pure form or as a forage component under industrial conditions [14].

Due to the ability to form endospores, which remain viable even under such extreme conditions as high and low temperatures, radiation, nonoptimal pH and pressure, and the presence of toxic chemicals, bacteria from the genus *Bacillus* are considered as promising for use as a basis for probiotic feed additives [15,16,17]. According to published data, probiotics based on *Bacillus* bacteria can be used for protection against intestinal and respiratory pathogens, dysbacteriosis prevention during antibiotic therapy, and improvement in food digestion and migration in the gastrointestinal tract [16,18,19]. Moreover, bacilliform strains are able to attach to intestine walls and competitively replace dangerous pathogens, such as *Escherichia coli* and *Staphylococcus aureus*.

*Bacillus* bacteria synthesize a number of antimicrobial compounds characterized by a selective activity against pathogens, but not beneficial microbiota [20,21,22]. Such antimicrobial metabolites have different mechanisms of action. For example, bacitracin A from *B. licheniformis* destructs a peptidoglycan layer of the bacterial cell wall. Bacilysin, which was first isolated from *B. subtilis* A14 [23], is active against a wide range of bacteria; for example, it inhibits *E. coli* growth with the MIC value of 0.001 μg/mL [24]. Mersacidin produced by *Bacillus* sp. HIL Y-85,54728 possesses the activity towards a number of Gram-positive bacteria including *S. aureus* SG511 (MIC value is 1 μg/mL) [25,26].

Bacillar metabolites can affect the lipid bilayer of a plasmatic membrane. For example, homopolymeric ε-poly-L-lysine from *B. subtilis* SDNS possesses antimicrobial activity towards Gram-positive and Gram-negative bacteria as well as fungi [27]. Using electrostatic interactions, this compound is attached to the phospholipid layer of the plasmatic membrane affecting membrane permeability and causing cell death.

Along with antimicrobial properties, *B. subtilis* produces enzymes utilizing the products of putrefactive decay of tissues, recovers populations of microorganisms composing the intestine normoflora, and synthesizes amino acids, vitamins, and immunoactive compounds. *B. licheniformis* produces a number of enzymes involved into food digestion and toxin neutralization and stimulates interferon production that results in a suppression of the growth and propagation of pathogenic microorganisms and viruses facilitating the normalization of intestine microflora [28,29,30,31,32].

Thus, the screening of new probiotic *Bacillus* strains as well as the improvement and stabilization of their antimicrobial properties by selection and chemical or physical mutagenesis represent relevant tasks. The purpose of this study was obtaining of *B. subtilis* and *B. licheniformis* strains with an improved antagonistic activity towards *E. coli* and *S. aureus* via UV mutagenesis coupled with selection as well as the experimental evaluation of the safety profile and probiotic potential of these strains for use as a basis of feed additives.

## 2. Materials and Methods

### 2.1. Microorganisms

Test cultures of *Escherichia coli* VKPM B-6645 and *Staphylococcus aureus* subsp. *aureus* VKPM B-6646 were obtained from a collection of the Laboratory of Biotechnology of Industrial Microorganisms of the Russian Biotechnological University (ROSBIOTECH, Moscow, Russia).

### 2.2. Media Composition for the Strain Maintenance and Morphological and Cultural Studies

*Bacillus* strains and test cultures of *E. coli* and *S. aureus* were maintained on the LA medium of the following composition (g/L): yeast extract, 5.0; meat peptone, 15.0; NaCl, 5.0; agar, 20.0 (pH 7.0–7.2); after one month of storage at 4 °C, cultures were reinoculated onto fresh medium. For long-term storage, cultures were freeze-dried using dried milk as a carrier.

The submerged fermentation of strains was carried out using the LB medium of the following composition (g/L): casein hydrolysate, 10.0; yeast extract, 5.0; NaCl, 10.0 (pH 6.8–7.0).

### 2.3. Strain Isolation and Identification

Strain isolation was performed according to Camacho et al. [33]. Soil (1 g) or milk (1 mL) samples were placed into tubes containing 9 mL of sterile physiological solution and thoroughly vortexed. Then, 1 mL of a solution was transferred into 250 mL flasks containing 50 mL of LB medium and incubated at 37 °C for 12 h. After sampling of 1 mL, the sample was transferred into tube with sterile distilled water, and a series of sequential dilutions was prepared. From each dilution variant, 0.1 mL was taken and inoculated onto Petri plates with agarized LA medium. Plates were incubated at 28 °C in the dark. The first colonies were observed after 24 h of incubation. Separate colonies were transferred onto Petri plates with fresh LA medium.

Identification of strains was carried out by a comparison of their morphological and biochemical traits with the published data [34]. Genetic identification of isolated strains and the derived mutants was performed at the “Bioengineering” Resource Sharing Center) of the Federal Research Center “Fundamentals of Biotechnology”.

DNA isolation was carried out according to Bulygina [35]; the concentration of the obtained DNA preparations was 30–50 μg/mL. To perform PCR and the further sequencing of the obtained PCR fragments of 16S rRNA genes, we used universal primer systems providing detection of both eubacteria (11f-1492r) and archaea (8fa-A915R). The amplification was carried out in 50 μL of the following reaction mix: 1× buffer for BioTaq DNA polymerase (17 mM (NH_4_)_2_SO_4_, 67 mM Tris-HCl (pH 8.8), 2 mM MgCl_2_); 12.5 nmoles of each dNTP; 50 ng of template DNA; 5 pmoles of each primer; and 3U BioTaq DNA polymerase enzyme (Dialat Ltd., Moscow, Russia). The first PCR cycle included denaturation for 9 min at 94 °C, annealing for 1 min at 55 °C, and elongation for 2 min at 72 °C and was followed by 30 cycles of denaturation for 1 min at 94 °C, annealing for 1 min at 55 °C, and elongation for 2 min at 72 °C; at the end of the process, a final extension for 7 min at 72 °C was performed.

PCR products were electrophoretically analyzed in 1% agarose at the electric field intensity of 6 V/cm. Isolation and purification of PCR products from agarose gel was carried out using a WizardPCRPreps kit (Promega, Madison, WI, USA) according to the manufacture’s recommendations. The obtained PCR fragments of genes encoding 16S rRNA were sequenced at the “Bioengineering” Resource Sharing Center of the Federal Research Center “Fundamentals of Biotechnology” using an Applied Biosystems 3730 genetic analyzer (ThermoFisher Scientific, Waltham, MA, USA) and a BigDyeTerminator v.3.1 cycle sequencing kit (ThermoFisher Scientific, Waltham, MA, USA) according to the manufacture’s recommendations. The sequencing was performed in both forward and reverse directions using amplifying and internal primers.

A phylogenetic analysis of the obtained nucleotide sequences of rRNA genes was carried out using a BLAST algorithm of the NCBI GenBank (http://www.ncbi.nlm.nih.gov, accessed on 8 August 2023). The alignment and editing of the obtained sequences were realized using a Bioedit program package (http://jwbrown.mbio.ncsu.edu/BioEdit/bioedit.html, accessed on 8 August 2023).

### 2.4. Cultivation of Bacillus Strains on a Liquid Nutrient Medium

To obtain culture broth, we used several liquid nutrient media of the following composition (g/L):

- Medium 1: soybean meal, 20.0; NaNO_3_, 3.0; K_2_HPO_4_, 1.0; MgSO_4_, 0.2; NaCl, 3.0;
- Medium 2: corn meal, 25.0; NaNO_3_, 3.0; K_2_HPO_4_, 1.0; MgSO_4_, 0.2; NaCl, 3.0;
- Medium 3: pea meal, 25.0; NaNO_3_, 3.0; K_2_HPO_4_, 1.0; MgSO_4_, 0.2; NaCl, 3.0;
- All three media were prepared using a tap water (pH 6.8–7.0).

To obtain inoculate, 10 mL of sterile 0.9% NaCl solution was added to the tube with the grown culture. The top agar layer was accurately scraped by a sterile inoculation loop, and a suspension was transferred into 750 mL flasks containing 100 mL of LB medium. The culture was then incubated for 24 h at 37 °C and 250 rpm using an Innova 44 shaker (New Brunswick, Germany). The resulting inoculate (10 vol. %) was transferred into fresh flasks with 100 mL of medium, which composition was determined by the aim of the experiment and cultivated for 24 h under the same conditions.

After completion of the incubation, culture broth was inactivated (if necessary) by heating on a water bath for 30 min at 80 °C.

### 2.5. Obtaining of a Dry Biomass of Bacillus Strains

Cultivated microbial biomass was separated from culture fluid by centrifugation at 4500 rpm during 40 min. Then, the culture broth was decanted, and the remaining biomass was freeze-dried using an ALPHA 2-4LD Plus freeze-dryer (Martin Christ Gefriertrocknungsanlagen GmbH, Osterode am Harz, Germany).

### 2.6. UV Mutagenesis of Bacillus Strains

After a 48 h growth, the biomass of a *Bacillus* culture was washed off with sterile water and filtered through a cotton filter to remove agar medium fragments. The spore concentration was calculated using a Goryaev–Thoma counting chamber (Graticules Optics, Tonbridge, UK) and adjusted to (1.5–2)·10^6^ spores/mL. The final suspension was placed under a short-wave (250–280 nm) Mineralight UV lamp (Analytikjena, Upland, CA, USA) at a distance of 40 cm. The exposure time varied from 5 to 30 min. A 0.1-mL aliquot of the treated suspension was inoculated onto Petri plate with agar medium and incubated for 48 h at 37 °C. The survival rate of colonies was determined as the percentage of colonies grown in the treated variants compared to the untreated control. The mutagenesis efficiency was determined by the percentage of morphologically changed colonies grown after the UV treatment.

After each UV treatment, 10–20 mutant colonies were selected for the further work based on changes in their color, form, or growth rate. Selected colonies were reinoculated onto fresh LA medium. Grown isolated colonies were then cultivated on the liquid Medium 1 to analyze their antagonistic, cellulolytic, and amylolytic activities. The most active strains were selected for the further mutagenesis/selection cycles. The total number of these cycles was 7.

### 2.7. Antagonistic Activity Assay

The antagonistic activity of probiotic strains was evaluated by the agar diffusion method using two test cultures (*E. coli* and *S. aureus*). Test cultures were grown on LA medium for 22 ± 2 h at 37 °C. The grown cultures were washed off from the agar surface with a sterile 0.9% NaCl solution, and concentration of microbial cells in the resulting suspension was adjusted to the standard turbidity sample (5 NTU). The obtained suspension was added to melted agarized LA medium, which temperature did not exceed 49 ± 1 °C, thoroughly mixed, and poured into sterile Petri plates. After solidification, 8 mm wells were made in the agar using a sterile borer of the corresponding diameter. Inactivated (see Section 2.4) culture broth of the studied probiotic strains was dropped into each well; in the case of a control, sterile water was used. After addition of culture broth, plates were left for 1–2 h at room temperature, then incubated for 20–24 h at 36 ± 1 °C, after which the growth inhibition zones were measured.

### 2.8. Evaluation of the Bacterial Growth Dynamics

Bacterial growth dynamics was determined spectrophotometrically. After sampling of culture broth aliquots, their optical density at 600 nm was measured using a spectrophotometer (ThermoFisher Scientific, Waltham, MA, USA). The number of formed spores was calculated in a Goryaev–Thoma counting chamber at 100× magnification using a Primo Star microscope (Carl Zeiss, Obercohen, Germany).

### 2.9. Cellulolytic Activity Assay

Cellylolytic activity of *B. subtilis* and *B. licheniformis* was evaluated according to Maslennikova et al. [36] with some modifications. Culture broth (5 μL) was inoculated onto Petri plates with agar medium of the following composition (g/L): NaNO_3_, 3.0; K_2_HPO_4_, 1.0; MgSO_4_, 0.5; KCl, 0.5; peptone, 0.2; agar, 17.0; microcrystalline cellulose (MCC) or carboxymethyl cellulose (CMC), 10.0. An equal volume (5 μL) of distilled water was used as the control. Plates were left for 24 h at 37 °C, then supplemented with 20–25 mL of a preliminarily prepared Lugol’s iodine solution (2.0 g of KI, 1.0 g of I, 0.1 L of distilled water); after 3–5 min, the excess liquid was removed. Cellulolytic activity of strains was evaluated by the presence and size (mm) of clearing zones around the points of a culture broth inoculation. All experiments were arranged in three replications.

### 2.10. Amylolytic Activity Assay

The amylolytic activity of the studied strains was evaluated according to Donkova et al. [37] with some modifications. A hot nutrient medium containing 1.5% agar and 1% starch paste was poured into Petri plates. After solidification, 5 μL of culture broth of the studied strain was placed onto the medium surface; the same volume of distilled water was used as a control. Plates were incubated for 24 h at 37 °C in a thermostat, then poured with 20–25 mL of the Lugol’s iodine solution diluted with distilled water (1:10), and the excess of the liquid was poured out. Medium was stained in a blue color excepting zones, where enzymatic cleavage of starch took place. The amylolytic activity was evaluated by the diameter (mm) of the clearing zone around the point of a culture broth inoculation. Each experimental variant included 3 Petri plates; the experiment was arranged in three replications.

### 2.11. Evaluation of Spore Resistance to Divverent pH Levels

Spore resistance to different pH levels was determined according to Haller et al. [38]. Spores of the studied strains were obtained by heating aliquots of a 2 day culture broth for 90 min at 60 °C to eliminate vegetative cells. The initial concentration of obtained spores was adjusted to 4 × 10^7^ CFU/mL. The obtained spore suspension was incubated in LB medium at 40 °C and different pH levels. The obtained culture broth was heated for 90 min at 60 °C; 0.1 mL of the broth was sampled and used to prepare a series of dilutions, which were inoculated on LA medium to obtain a lawn culture and calculate the CFU content. The remaining spore suspension was concentrated by centrifugation (10 min at 6000 rpm). After removal of a supernatant, the residue was transferred to the equal volume of fresh LB medium (pH 3.0), and left for 90 min. Then, the spore suspension was heated again for 90 min at 60 °C, and 0.1-mL aliquot was sampled to determine the CFU concentration. The remaining spore suspension was concentrated by centrifugation as described above, transferred into the Medium 1 (pH 7.0), and incubated for 150 min. The obtained culture broth was heated again as described above, and the next 0.1 mL aliquot was sampled to determine the CFU concentration. All experiments were arranged in three replications.

The CFU concentration was calculated using the following formula:

(1) $$X=\frac{A\times 10^{n}}{a}\times 10\;,$$

where *X* is the number of CFU per 1 g or 1 mL of a biomass, *A* is the mean arithmetic number of colonies grown in three Petri plates, 10*^n^* is the dilution degree, and *a* is the weight of the dry biomass or the volume of the aliquot used for inoculation.

### 2.12. Determination of the Viability of Bacillus Cells after Exposure to Organic Acid Solutions

Dry *Bacillus* biomass (5 g) was transferred into a flask with 45 mL of distilled water acidified by one of the chosen organic acids (acetic, ascorbic, formic, or citric) to pH 3. After thorough mixing, the flask was incubated for 3 or 24 h at 40 °C. The number of viable bacterial cells was determined by the inoculation of the corresponding sample dilution onto agarized LA medium. A suspension of a dry microbial biomass in distilled water with neutral pH was used as the control. The CFU content was determined using Formula (1). Each experimental variant included 3 Petri plates; the experiment was arranged in three replications.

### 2.13. Determination of Spore Resistance to High Temperatures

Dry bacterial biomass (1 g) was aseptically transferred into a tube with 9 mL of sterile physiological solution, thoroughly mixed, and heated for 5–20 min at 80 and 100 °C using a water bath. Then, a series of sequential dilutions was prepared and inoculated onto Petri plates with LA medium. For each dilution variant, three plates were inoculated to calculate arithmetical mean. The experiment was arranged in three replications. The number of viable cells was calculated using Formula (1).

### 2.14. Antibiotic Sensitivity Assay

An aliquot (5 g) of a dry biomass was aseptically transferred into a sterile 250 mL flask containing 50 mL of a sterile physiological solution and mixed for 30 min to obtain a homogenous suspension, which was used to prepare a tenfold dilution. Then, 100 μL of the resulting suspension were transferred into Petri plates with agarized LA medium and thoroughly spread over the agar surface using a sterile spatula. Then, sterile paper disks impregnated with antibiotics were placed into each plate (4 disks per plate). After 24 h of incubation, growth inhibition zones were evaluated.

### 2.15. Statistical Data Treatment

The data shown herein represent the arithmetic means of values from three or more replications (depending on the assay in question). The statistical treatment of data was carried out using a MS Excel 2016 statistic software package and standard methods for determination of the t-test criterion at 5% significance level. The significance of difference between different samplings was determined using the least significant difference method.

## 3. Results

### 3.1. Strain Isolation and Identification

Due to the performed cultural and morphological examination, two groups of bacteria, *B. subtilis* and *B. licheniformis*, were isolated. The initial selection of isolated strains was based on their antagonistic activity towards test cultures of *E. coli* and *S. aureus*. As a result, two strains able to suppress the growth of these test cultures were revealed, *B. subtilis* RBT-7 and *B. licheniformis* RBT-11.

In the case of *B. subtilis* RBT-7, almost complete (1490 bp) sequence of the bacterial component of the amplificate of the gene encoding 16S rRNA was determined (see Figure S1). No minor components were detected in sample spectrograms that evidenced the purity of the analyzed material. A taxonomical identification showed the strain belonged to the genus *Bacillus* with the maximum similarity (99.93%) to the reference *B. subtilis* strain NCIB 3610.

For *B. licheniformis* RBT-11, reliable sequence (1312 bp) of the bacterial component of the amplificate of the gene encoding 16S rRNA was determined (see Figure S2). No archaeal components were revealed. The level of similarity with the closest reference strain (*B. licheniformis* ATCC 14580) was 100%.

Thus, taking into account micro- and macromorphology of strain colonies as well as data on their genetic identification, we concluded they belonged to *B. subtilis* and *B. licheniformis* species.

### 3.2. Improvement of the Target Activity of B. subtilis and B. licheniformis

**Figure 1:**
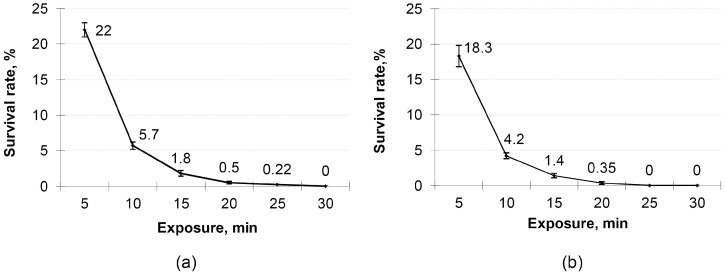
Survival rate of (**a**) *Bacillus subtilis* B-7 and (**b**) *B. licheniformis* B-11 under different exposures to UV radiation.

The improvement in the antagonistic activity of isolated strains was achieved by UV mutagenesis. To select the optimal exposure duration, cells of both strains were treated with UV radiation with a varying exposure range (5–30 min) with a 5 min interval. The results of this experiment are shown in Figure 1.

Since the survival rate for the optimal mutagenesis process should fall within the range of 0.1–1.0%, the optimal exposure time for *B. subtilis* B-7 and *B*. *licheniformis* B-11 was 25 and 20 min, respectively. The further strain improvement process included several stages. Among colonies grown from radiated cells, we registered those morphologically differing from initial strains in relation to the colony color (from cream to white), size (larger or smaller than the colony size of initial strains), edge shape (wavy or even), and consistency (slimy, sticky, or rough).

For each mutagenesis step, each obtained and selected bacterial isolate was evaluated first for the presence of a higher antagonistic activity towards test cultures and then for the presence of higher cellulolytic and amylolytic activity compared to the parental strain. The choice of *E. coli* and *S. aureus* as test strains for evaluation of the antagonistic activity of the parental and mutant strains was based on the recommendations of the European Food Safety Authority (EFSA), which proposed the “Qualified Presumption of Safety” (QPS) concept applied to certain groups of microorganisms used in feed and food additives [39].

During the study we found that only *B. subtilis* was able to hydrolyze MCC (CMC); no such activity was observed for *B. licheniformis*. The amylolytic activity of the finally derived strains 2.2-fold exceeded that of parental ones. Thus, the performed 7-step UV mutagenesis with the further selection resulted in two mutant strains, *B. subtilis* RBT-7/32 and *B. licheniformis* RBT-11/17.

Since bacilli do not have too many distinctive morphological traits, the taxonomic belonging of both mutants to the corresponding genera was examined by the analysis of their 16S rRNA sequences compared to those of parental strains. The identity of these sequences in parental and mutant strains confirmed their belonging to *B. subtilis* and *B. licheniformis*, and the fact that mew mutations did not involve the corresponding region of 16S rRNA.

A comparison of averaged parameters characterizing antagonistic, cellulolytic, and amylolytic activities of both parental and mutant strains is shown in Table 1. The performed analysis for determination of the least significant difference between the values obtained for parental and mutant strains at *p* < 0.05 revealed a significant difference between the strains.

### 3.3. Growth of B. subtilis RBT-7/32 and B. licheniformis RBT-11/17 Strains on Different Substrates

The growth of the obtained mutant strains was studied on media containing some components of forage for livestock and poultry (soybean, pea, and corn meals); the LB medium was considered as a control. According to the performed statistical analysis, no significant difference (*p* < 0.05) in the strain productivity was found between the studied medium variants in relation to the bacterial biomass (Table 2), though the best result for both mutant strains was obtained for medium supplemented with soybean meal (20 g/L, Medium 1).

### 3.4. Evaluation of the Spore Germination of Mutant Strains at Different pH Levels

The in vitro imitation of the transit of *B. subtilis* RBT-7/32 and *B. licheniformis* RBT-11/17 spores through a gastrointestinal tract of chickens was performed in LB medium at different pH and exposure times. The results are shown in Table 3.

The initial spore concentration was 4 × 10^7^ CFU/mL. After a 1 h incubation at pH 5.0, the value of this parameter in *B. subtilis* RBT-7/32 and *B. licheniformis* RBT-11/17 was 3.3 × 10^7^ and 3.0 × 10^7^ CFU/mL, respectively, which indicates the ability of spores to transit to vegetative forms under such conditions. In the course of the further 1.5 h incubation at pH 3.0, the concentration of RBT-7/32 and RBT-11/17 spores decreased to 2.2 × 10^7^ and 2.0 × 10^7^ CFU/mL, respectively. The next incubation step (pH 7.0 for 2.5 h) did not result in any changes in the spore concentration; for both strains, it remained at the level of 2.0 × 10^7^ CFU/mL. Therefore, we can suppose that spores of both strains should keep the ability to germinate into vegetative structures even in the case of passage through a gastrointestinal tract thus confirming their probiotic properties. No significant difference (*p* < 0.05) was revealed between the data obtained for the parental and mutant strains.

### 3.5. Evaluation of the Heat Tolerance of Mutant B. subtilis and B. licheniformis Strains

The ability to remain viable after high-temperature heating is an important technical characteristic of probiotic forage additives. The results of the corresponding evaluation are shown in Table 4. According to these data, 15–20 min heating of the water suspension of the dry biomass of both studied strains at the chosen temperatures did not provide a significant effect on the survivability of their spores. No significant difference (*p* < 0.05) was revealed between the data obtained for the parental and mutant strains, though the last ones showed better heat tolerance.

### 3.6. Antibiotic Sensitivity of Mutant B. subtilis RBT-7/32 and B. licheniformis RBT-11/17 Strains

The results of the study of the sensitivity of mutant strains to different antibiotics are presented in Table 5. According to these data, strains were resistant to bacitracin (100 U), colistin sulfate (10 μg/mL), virginiamicin (100 μg/mL), tylosin (20 μg/mL), tiamulin (300 μg/mL), benzylpenicillin (10 U), and polymyxin (300 μg/mL), moderately resistant to doxicycline (30 μg/mL) and tylosin (200 μg/mL), and sensitive to tetracycline (30 μg/mL), furazolidone (300 μg/mL), amoxicillin (25 μg/mL), lincomycin (15 μg/mL), erythromycin (15 μg/mL), gentamycin (120 μg/mL), kanamycin (30 μg/mL), ampicillin (10 μg/mL), enrofloxacin (5 μg/mL), florfenicol (30 μg/mL) and furazolidone (300 μg/mL) taken at the studied concentrations. The performed statistical treatment showed no significant difference (*p* < 0.05) between the data obtained for the parental and mutant strains, thus confirming the mutations did not involve genes responsible for the antibiotic resistance.

Since one of the future applied tasks included the development of a feed additive composed of two probiotic strains, we also examined any possible changes in the sensitivity (i.e., size of the growth inhibition zone) of both strains in the case of their mixing at a ratio of 1:1. The results of this experiment are shown in Table 6; no significant difference between the mixes of parental or mutant strains in relation to the antibiotic sensitivity was observed (*p* < 0.05).

### 3.7. Survivability of Mutant B. subtilis RBT-7/32 and B. licheniformis RBT-11/17 Strains in Organic Acid Solutions

The results of examination of the survivability of both parental and mutant strains in solutions of different organic acids are shown in Table 7. Incubation of a dry biomass of probiotic strains suspended in water acidified with various organic acids did not influence their viability.

## 4. Discussion

New approaches developed in recent years for prevention and treatment of gastrointestinal diseases are based on the recovery of the natural microflora by probiotic preparations [40,41,42,43]. *Bacillus* bacteria represent promising strains for the development of probiotic additives; their probiotic activity is based on the synthesis of antimicrobial compounds, enhancement of a nonspecific and specific immunity, stimulation of the growth of the normal intestinal microflora, and production of digestive enzymes. Therefore, a search of microorganisms, which possess the above-mentioned properties, for the development of highly effective probiotic feed additives, represents an important applied task [44].

Today an improvement in *Bacillus* strains in terms of an increased biosynthesis of a target product is widely realized by molecular genetic methods [45,46]. However, genetic engineering methods are not suitable for the development of strains used in feed additives because of the existing legislative restrictions for use of genetically modified microorganisms. Moreover, genetically engineered strains are often reported to be unstable in terms of the productivity and maintenance of newly acquired traits [47,48]. Introduction of alien genes or promoters may result in a more intensive use of intracellular resources, resulting in an additional metabolic burden on the host; moreover, alien genes may encode components that are toxic to the host cell. Therefore, the modified strain can obtain a fitness advantage by losing or inactivating such genes [48]; in this case, such a mutant can rapidly spread its genotype over the whole population causing the worsening or even a complete loss of the introduced trait in the entire population that may drastically influence on the possibility of the further industrial use of the strain. In addition, the use of genetically engineered organisms is limited by consumer acceptance issues.

Thus, in most cases, the main efforts to improve strains promising for industrial application are based on natural strategies, such as random mutagenesis. The resulting mutants are usually much more stable, and their use is not restricted by legislation or public opinion. To date, there are many examples of strains with good probiotic potential obtained by UV mutagenesis (see, for example, [49,50]). These reasons substantiated the choice of the UV mutagenesis with the further selection as the method for improvement in the antagonistic activity of *B. subtilis* RBT–7 and *B. licheniformis* RBT–11 strains. In addition, the obtained mutant strains did not carry any genetic modifications that excluded the risk of a possible transfer and spreading of new genetic information. The repeated 16S rRNA analysis revealed the identity of nucleotide sequences of both parental and mutant strains, so mutations provided the achieved improvement in antagonistic properties of both strains were not associated with the 16S rRNA gene.

Selection of mutant isolates is an important stage in the strain improvement process. Of course, this method is based on a kind of subjectivism; nevertheless, correlation between changes in the productivity level and changes of morphological traits of a strain has been confirmed by a number of studies [51]. In our study, we obtained mutant *B. subtilis* RBT-7/32 and *B. licheniformis* RBT-11/17 strains, which ability to suppress the growth of *E. coli* exceeded that of parental strains by 77 and 63%, respectively, and the ability to suppress the growth of *S. aureus* exceeded that of parental strains by 80 and 79%, respectively. An increased suppression of *E. coli* and *S. aureus* by new mutant strains can be explained by several factors. First, the growth rate of new strains may increase compared to the parental strains; second, mutant strains may enhance biosynthesis of compounds possessing antibiotic properties [52]. Since no difference between the growth rate of parental and mutant strains was observed, the observed effect is probably associated with the enhanced biosynthesis of antimicrobial compounds (bacteriocins and antibiotics). However, according to the EFSA guidance [39], these antibiotics should differ from those used in medicine and veterinary. Therefore, the nature of these compounds should be elucidated in the further studies of probiotic properties of *B. subtilis* RBT-7/32 and *B. licheniformis* RBT-11/17 strains.

The problem of incapacity of monogastric animals (chickens and pigs) to digest and assimilate highly structured forage components can be solved by addition of the corresponding enzymes into their ration. An alternative solution is the development of bacterial preparations based on microorganisms synthesizing extracellular hydrolytic enzymes, which provide better assimilation of forage components. In view of this fact, we selected the obtained mutant strains not only by their antagonistic properties, but also by their cellulolytic activity. One of the resulting strains, *B. subtilis* RBT-7/32, was capable of more efficient (by 48 and 51%) utilization of MCC and CMC than the parental strain. The second strain, *B. licheniformis* RBT-11/17, as well as its parental strain, were not able to hydrolyze MCC and CMC. Both parental strains demonstrated an amylolytic activity, which was improved by 42 and 40% in mutant *B. subtilis* RBT-7/32 and *B. licheniformis* RBT-11/17 strains.

One of the main advantages of preparations based on *Bacillus* strains is the ability of these microorganisms form spores [18,53]. This property provides some technological advances for the introduction of additives into a forage, since spores remain stable during long-term storage, steaming, and granulation. The performed evaluation of the thermal stability showed the spore heating up to 80 and 100 °C did not provide any negative effect on their viability. In the case of the maximum impact (heating to 100 °C for 20 min), the number of CFU of *B. subtilis* RBT-7/32 and *B. licheniformis* RBT-11/17 was 4.2 × 10^11^ and 3.7 × 10^11^ that was not significantly lower than the initial concentrations (5.2 × 10^11^ and 4.8 × 10^11^, respectively).

There are several reasons for the use of low-molecular organic acids (formic, propionic, acetic, etc.) in husbandry. Forage and water represent the main sources of pathogenic microorganisms infecting poultry [54]. For example, 75–88% of water used in poultry farming is significantly contaminated with bacteria, such as *E. coli*, *Campilobacter*, *Pseudomonas*, etc. Note that the above-mentioned pathogenic microorganisms often are highly resistant to common antibacterial preparations [55,56]. Therefore, use of acidifying agents makes it possible to disinfect water due to suppression of a pathogen microflora as well as to normalize the acid-base balance in the intestine providing favorable conditions for the growth of the intestine normoflora and biosynthesis of enzymes facilitating digestion and nutrient assimilation [56,57,58]. Since introduction of probiotic preparations with water is one of the possible ways of their delivery, the survival of *B. subtilis* RBT-7/32 and *B. licheniformis* RBT-11/17 in solutions of organic acids at pH 3.0 is also important. In our study, treatment of a dry biomass of bacterial cells with solutions of citric, ascirbic, formic, and acetic acids for 3 and 24 h at 40 °C did not provide any negative effect on the spore ability to form vegetative cells.

In spite of all advantages of probiotic use, a complete rejection of antibiotic preparations for the treatment of some diseases is impossible and inexpedient from the economic point of view. Therefore, investigation of the possibility to overcome the existing antibiotic resistance of microorganisms is very important. One of possible research directions is a study of the antibiotic resistance of probiotic microbes, since the use of such strains combined with antibiotics for a complex therapy of a number of diseases may allow researchers to develop novel treatment regimens [59]. Nevertheless, to prevent the spreading of antibiotic resistance, living microorganisms used in antibiotic-resistant feed additives should not become a source of acquired antibiotic resistance determinants for bacteria inhabiting the gastrointestinal tracts of humans and animals.

In view of this concept, the study of the resistance of the RBT-7/32 and RBT-11/17 strains to common veterinary antibiotics with the determination of minimal inhibiting concentrations (MIC) was of some interest. Note that in the course of the study of the antibiotic tolerance of new strains, we showed (according to the EFSA recommendations) that neither nutrient medium composition and pH, nor temperature of cultivation influenced on the susceptibility of both strains to antibiotics.

EFSA experts proposed a special antibiotic panel to evaluate the safety profile of microorganisms used in probiotic preparations [60]. The data on the resistance or susceptibility of strains to the compounds included in this panel allow researchers to forecast the resistance or sensitivity of these strains to the antibiotic preparations included into the corresponding groups. In our study, we did not use the whole list of recommended antibiotic compounds. Nevertheless, the performed antibiotic sensitivity tests showed that both parental and new strains of *B. subtilis* and *B. licheniformis* were sensitive to antibiotics included to the EFSA 14 list [60] (ampicillin, gentamycin, kanamycin, erythromycin, and tetracycline). These results confirm the studied probiotic strains meet the criterion proposed by EFSA, namely, sensitivity to at least two antibacterial preparations used in clinical practice.

Based on the obtained data, we also made a conclusion about resistance of our probiotic strains to antibiotic preparations taken in the studied concentrations and belonging to the following classes: polypeptides (bacitracin), macrolides (tilosin, tiamulin) and macrocyclic antibiotics (virginiamycin), lactams (benzylpenicillin), and polymyxins (polymyxin). Note that such antibiotics as tilosin, tiamulin, and virginiamycin are used in agriculture [59,61,62]. Therefore, the revealed antibiotic resistance of *B. subtilis* RBT-7/32 and *B. licheniformis* RBT-11/17 strains makes it possible a combined application of these antibiotics with probiotic preparations based on these two strains. This, in turn, may improve the therapeutic effect of antibiotics and reduce their negative impact on the intestine microbiocenosis of livestock and poultry [63].

Thus, the performed study resulted in the development of two strains, *B. subtilis* RBT-7/32 and *B. licheniformis* RBT-11/17, possessing an improved antagonistic activity towards *E. coli* and *S. aureus* test cultures. The data on the thermal stability of these probiotic strains, their ability to grow within a wide acidity range, resistance to antibiotics, and the ability to synthesize enzymes improving digestion processes make it possible to conclude that they are promising for the development of a feed additive based on these strains.

## Figures and Tables

**Table 1:** Comparison of the basic parameters characterizing target activities of parental and mutant *B. subtilis* and *B. licheniformis* strains.

Parameter	Strains			
	*B. subtilis* B-7	*B. subtilis* RBT-7/32	*B. licheniformis* RBT-11	*B. licheniformis* RBT-11/17
Diameter of the *E. coli* growth inhibition zone, mm	12.4 ± 0.1	22.0 ± 0.2	11.3 ± 0.2	18.5 ± 0.1
Diameter of the *S. aureus* growth inhibition zone, mm	11.7 ± 0.1	20.8 ± 0.1	10.8 ± 0.1	19.4 ± 0.1
Diameter of the MCC hydrolysis zone, mm	10.6 ± 0.2	20.3 ± 0.1	-	-
Diameter of the CMC hydrolysis zone, mm	11.0 ± 0.1	22.7 ± 0.1	-	-
Diameter of the starch hydrolysis zone, mm	12.5 ± 0.1	21.6 ± 0.1	11.8 ± 0.1	19.7 ± 0.2

**Table 2:** Optical density (OD_600_) of parental and mutant *B. subtilis* and *B. licheniformis* strains grown on different nutrient media.

Medium	Optical Density OD_600_, Rel. Units			
	*B. subtilis* RBT-7	*B. subtilis* RBT-7/32	*B. licheniformis* RBT-11	*B. licheniformis* RBT-11/17
LB medium	2.2 ± 0.13	3.34 ± 0.21	2.0 ± 0.13	3.04 ± 0.15
Medium 1 (soybean meal)	1.6 ± 0.21	2.98 ± 0.34	1.5 ± 0.15	2.86 ± 0.25
Medium 2 (pea meal)	1.5 ± 0.15	2.67 ± 0.22	1.3 ± 0.1	2.70 ± 0.18
Medium 3 (corn meal)	1.2 ± 0.12	2.36 ± 0.53	1.2 ± 0.12	2.25 ± 0.40

**Table 3:** Survivability of *B. subtilis* and *B. licheniformis* spores under different conditions.

Conditions (pH Value and Exposure Time)	Spore Concentration, CFU/mL			
	*B. subtilis* RBT-7	*B. subtilis* RBT-7/32	*B. licheniformis* RBT-11	*B. licheniformis* RBT-11/17
Control (initial point)	4.0 × 10^7^	4.0 × 10^7^	4.0 × 10^7^	4.0 × 10^7^
pH 5.0, 1 h	3.0 × 10^7^	3.3 × 10^7^	3.2 × 10^7^	3.0 × 10^7^
pH 3.0, 1.5 h	2.1 × 10^7^	2.2 × 10^7^	1.8 × 10^7^	2.0 × 10^7^
pH 7.0, 2.5 h	2.0 × 10^7^	2.0 × 10^7^	1.7 × 10^7^	2.0 × 10^7^

**Table 4:** Spore survivability of parental and mutant *B. subtilis* and *B. licheniformis* strains under different temperature regimes.

Temperature, °C	Exposure, min	Spore Concentration, CFU/mL			
		*B. subtilis* RBT-7	*B. subtilis* RBT-7/32	*B. licheniformis* RBT-11	*B. licheniformis* RBT-11/17
-	-	5.0 × 10^11^	5.2 × 10^11^	4.0 × 10^11^	4.8 × 10^11^
80	10	4.8 × 10^11^	5.2 × 10^11^	3.7 × 10^11^	4.7 × 10^11^
	15	4.0 × 10^11^	5.1 × 10^11^	3.2 × 10^11^	4.7 × 10^11^
	20	3.4 × 10^11^	4.4 × 10^11^	3.0 × 10^11^	4.2 × 10^11^
100	10	4.0 × 10^11^	4.8 × 10^11^	3.2 × 10^11^	4.3 × 10^11^
	15	3.6 × 10^11^	4.6 × 10^11^	2.6 × 10^11^	4.0 × 10^11^
	20	3.2 × 10^11^	4.2 × 10^11^	2.2 × 10^11^	3.7 × 10^11^

**Table 5:** Antibiotic sensitivity of parental and mutant *B. subtilis* and *B. licheniformis* strains.

Antibiotic Group	Antibiotic	Dose, µg	Growth Inhibition Zone, (±0.2 mm) ^1^			
			*B. subtilis* RBT-7	*B. subtilis* RBT-7/32	*B. licheniformis* RBT-11	*B. licheniformis* RBT-11/17
Tetracyclines	Doxycycline	30	11	10	8	8
	Tetracycline	30	16	15	15	15
Polypeptides	Bacitracin	0.04 U	0	0	0	0
		10 U	0	0	0	0
		100 U	0	0	0	0
	Colistin sulfate	10	0	0	0	0
Nitrofurans	Furazolidone	300	14	14	15	15
Penicillins	Amoxicillin	25	24	23	24	22
Lincosamides	Lincomycin	15	23	23	21	22
Streptogramins	Stafac 110 (Virginiamicin)	50	11	10	10	10
		100	0	0	0	0
Macrolides	Tylosin	200	0	0	0	0
		20	10	10	10	10
	Tiamulin	300	0	0	0	0
	Erythromycin	15	26	25	25	24
Aminoglycosides	Gentamycin	120	24	22	22	22
	Kanamycin	30	22	20	22	21
Lactams	Ampicillin	10	12	12	12	12
	Benzylpenicillin	10 U	0	0	0	0
Quinolones	Enrofloxacin	5	30	30	26	26
Amphenicols	Florfenicol	30	26	25	25	25
Polymyxins	Polymyxin	300	0	0	0	0
Nitrofurans	Furazolidone	300	15	14	15	15

^1^ Size of inhibition zone: 0, antibiotic-resistant; ≤12 mm, moderately resistant; >12 mm, sensitive strain.

**Table 6:** Antibiotic sensitivity of parental or mutant *B. subtilis* and *B. licheniformis* strains inoculated in the form of a freeze-dried mix (at a ratio of 1:1).

Antibiotic Group	Antibiotic	Dose, µg	Growth Inhibition Zone, mm	
			*B. subtilis* RBT-7/ *B. licheniformis* RBT-11	*B. subtilis* RBT-7/32 *B. licheniformis* RBT-11/17
Tetracyclines	Doxycycline	30	10	9
Polypeptides	Bacitracin	10 U	0	0
Lactams	Ampicillin	10	12	11
Streptogramins	Stafac 110 (Virginiamicin)	100	0	0
Macrolides	Tylosin	200	0	0
	Tiamulin	300	0	0
Lactams	Benzylpenicillin	10 U	0	0
Polymyxins	Polymyxin	300	0	0

**Table 7:** Survivability of parental and mutant *B. subtilis* and *B. licheniformis* strains after incubation in organic acid solutions (pH 3.0, 40 °C).

Organic Acid	Exposure Time, h	Spore Concentration, CFU/mL			
		*B. subtilis* RBT-7	*B. subtilis* RBT-7/32	*B. licheniformis* RBT-11	*B. licheniformis* RBT-11/17
Control (water)	3	5.00 × 10^11^	5.10 × 10^11^	5.10 × 10^11^	5.20 × 10^11^
	24	4.80 × 10^11^	5.00 × 10^11^	5.00× 10^11^	5.18 × 10^11^
Citric acid	3	5.00 × 10^11^	5.14 × 10^11^	5.20 × 10^11^	5.14 × 10^11^
	24	4.82 × 10^11^	5.11 × 10^11^	4.92 × 10^11^	5.05 × 10^11^
Ascorbic acid	3	4.82 × 10^11^	5.23 × 10^11^	4.80 × 10^11^	5.10 × 10^11^
	24	4.80 × 10^11^	5.20 × 10^11^	4.75 × 10^11^	5.00 × 10^11^
Acetic acid	3	5.03 × 10^11^	5.03 × 10^11^	5.03 × 10^11^	5.03 × 10^11^
	24	5.00 × 10^11^	5.12 × 10^11^	4.90 × 10^11^	5.12 × 10^11^
Formic acid	3	5.10 × 10^11^	5.00 × 10^11^	5.00 × 10^11^	5.17 × 10^11^
	24	5.00 × 10^11^	5.14 × 10^11^	4.90 × 10^11^	5.00 × 10^11^

## Data Availability

The authors declare that the data supporting the findings of this study are available within the main text of the manuscript and supplementary materials. Raw data are available from the corresponding author upon reasonable request.

## Supplementary Materials

The following supporting information can be downloaded at: https://www.mdpi.com/article/10.3390/microorganisms11112729/s1, Figure S1: Sequence of the bacterial component of the 16S rRNA gene of *Bacillus subtilis* RBT-7. Figure S2: Sequence of the bacterial component of the 16S rRNA gene of *Bacillus licheniformis* RBT-11.
